# Pornography, sexual orientation and ambivalent sexism in young adults in Spain

**DOI:** 10.1186/s12889-024-17853-y

**Published:** 2024-02-05

**Authors:** Belén Sanz-Barbero, J. Francisco Estévez-García, Raquel Madrona-Bonastre, Gemma Renart Vicens, Laura Serra, Carmen Vives-Cases

**Affiliations:** 1 Department of Epidemiology and Statistics National School of Public Health, Instituto de Salud Carlos III, Madrid, Spain; 2 Consortium for Biomedical Research in Epidemiology & Public Health (CIBERESP), Madrid, Spain; 3 Department of Sociology II, Alicante University, Alicante, Spain; 4 Public Health Research Group, Department of Community Nursing, Preventive Medicine and Public Health and History of Science, Alicante University, Crta. San Vicente, S/N, 03690 Alicante, San Vicente del Raspeig Spain; 5 Research Group On Statistics, Econometrics and Health (GRECS), University of Girona, Girona, Spain

**Keywords:** Hostile sexism, Benevolent sexism, Pornography, Sexual orientation, Sex

## Abstract

**Background:**

On line platforms offer access to an almost unlimited variety of pornographic material that shows high levels of sexism. Despite this fact, there are still few studies that assess the effect of pornography on sexism in young adults The aim of this study is to analyze the association of pornography consumption and sexual orientation with benevolent sexism (BS) and hostile sexism (HS) in young men and women.

**Methods:**

We surveyed 2,346 people aged 18–35 years old. Multiple regression models were carried out for BS and HS. The independent variables: current pornography consumption and sexual orientation. Covariates: socio-demographic variables -age, sex, level of education and place of birth-.

**Results:**

*A) HS*: Men who consumed pornography had higher median values of HS than those who did not [β(95%CI):2.39(0.67;4.10)]. Homosexual/ bisexual men displayed lower values of HS than heterosexual men [β(95%CI):-2.98(-4.52;-1.45)]. The increase in HS levels associated with pornography consumption was notably greater in homosexual and bisexual women relative to heterosexual women, where that pattern was not observed [β(95%CI for interaction): 2.27(0.11; 4.43)]. B*) BS*: Mean values of BS were observed to be lower for both women [β(95%CI):-2.16(-2.99;-1.32)] and men [β(95%CI):-4.30(-5.75;-2.86)] who consumed pornography compared to those who did not. Homosexual/bisexual men recorded mean values of BS lower than heterosexual men [β(95%CI):-3.10(-4.21;-1.99)].

**Conclusions:**

Pornography consumption is related to sexism and differs according to sex and sexual orientation. As sexism is the substratum of inequality between men and women, it is urgent to launch affective-sexual education programs for young people that take into account the determinants of sexism.

## Background

Digital platforms offer easy, free and anonymous access to an almost unlimited variety of sexually explicit material that eroticizes inequality and violence against women [1–3]. Studies carried out on the most consumed pornographic content mention sexist pornography focused on male pleasure, where women are objectified and their sexuality is dependent on their ability to give pleasure to men [4]. Pornography eroticizes humiliating women, submission, lack of consent and, in short, violence against women [2, 4, 5]. According to the last report published by Pornhub, one of the most used pornographic websites in the world, in 2021, an average of 130 million visits per day was recorded; that is 51% more than the visits in 2016. In said year, 79% of the visits originated from the United States. Spain, which is the geographical area of this study, counted for 26% of the visits. People who visited the website from Spain in 2021 were 62% men; of which 26% were between 18 and 24 years [6].

Beyond the studies and reports that analyze visits to pornographic websites, prevalence studies show a high use of pornography in both the adolescent and young adult population [7]. The systematic review by Peter and Valkenburg [8] showed that an intentional use of pornography at some point in life ranging from 41% among Taiwanese adolescents [9] to prevalences greater than 80% in Germany (98% in boys; 81% in girls) [10]. In Spain, intentional pornography consumption in the last month in young adults (18-34) reached 47.5% being more than three times higher in men (56.7%) than in women (17.2%). This percentage decreases among the population of adults between 54 and 75 years of age to 25.6% [11]. In all cases, pornography consumption is higher in men than women [8, 11–13]. Recent studies have shown greater lifetime pornography consumption among lesbian/bisexual women (89.9%) than heterosexual women. (69.2%) [14]. This study identifies an association between orientation and pornography consumption in women, but not in men [14].

Despite the fact that pornography shows high levels of sexism, there are still few studies that assess the effect of pornography on sexism in young adults [15–17]. Glick and Fiske (1996) conceptualized the theory of ambivalent sexism (AS) with two subcomponents: hostile sexism (HS) and benevolent sexism (BS) [18]. HS includes openly negative attitudes towards women. It establishes a relationship of domination and control over women and emphasizes differences between men and women, forming a hierarchy that depreciates women when compared to men. BS is more subtle and is expressed with an affective tone. It considers women to be fragile beings that need to be protected by men and attributes stereotypes aligned with traditional hegemonic femininity and gender roles [18]. Both roles of AS (BS and HS) include three components: paternalism, differentiation between men and women according to gender roles and heterosexual intimacy. Heterosexual intimacy in BS casts a romantic idea on women that complements men in their search for happiness. In HS, this component is portrayed in terms of heterosexual hostility and expresses the perception that women use their sexuality as a way to dominate men [19].

AS by means of BS and HS permeates the social structure establishing an interdependence between both sexes while maintaining inequalities between men and women. AS establishes a masculine heterosexual domination in society that not only discriminates women, but also expresses hostility and discrimination towards non-heterosexual people [20]. Currently, there is consolidated evidence of the association of sex with AS, with men being more sexist than women [21–23], but information on the association between sexual orientation and AS is still scarce and heterogeneous [24–26].

The few studies that approach the relation between sexism and pornography consumption are not conclusive. While some demonstrate a positive correlation between AS and pornography consumption in men and women [15, 16], other studies have reported no significant associations between pornography consumption and sexism [17, 27, 28], and some have reported contrary results [29] showing that greater pornography consumption was associated with less sexist attitudes after controlling for participants´ sex.

The aim of this study is to analyze the association of pornography consumption and sexual orientation with HS and BS in young men and women. This study is based on the following hypotheses: A) There is an association between sexual orientation and BS/HS. This association is different in men and women; B) There is an association between pornography consumption and BS/HS. This association can be modified according to sex and sexual orientation.

## Methods

### Population and sample

Cross-sectional study of the Sexual Violence in Young People Survey carried out online with young men and women aged between 18 and 35, residing in Spain. The sample was calculated using the most recent data on the prevalence of sexual violence (SV) in Spain (lifetime prevalence of SV: 13.7%), published in the Macro Survey on Violence against Women [30], corrected for sex. Data on the Spanish population aged between 18 and 35 were obtained from the Spanish Statistical Office [31]. The minimum sample size needed was estimated at 2,500 questionnaires with quotas by sex, age, region and country of origin, in order to guarantee a sample error of ± 5%, considering a 95% confidence interval and prevalence estimates with a precision level of ( ±) 0.9. The sampled individuals were obtained from a probability-based, online, closed panel of adults aged 16 or older designed to be fully representative of the non-institutionalized Spanish civilian population. The panel is comprised of 138,393 active panel members. Panel members were recruited by single-use, individual invitations. They received only non-survey specific incentives through a points-based reward program. After carrying out a first pilot study (September 30- October 1, 2020) data collection was carried out between October 15 and October 28, 2020. A database of 2,525 individuals was obtained that, once refined,—resulted in 2,346 registrants.

Participation was voluntary and required a signed informed consent document. The study was conducted according to the guidelines of the Declaration of Helsinki and was approved by the Ethics Committee of the University of Alicante (ref. UA-2020–07-07).

### Variables

#### Dependent variables

BS and HS: In order to measure both AS variables, the Spanish version of the Ambivalent Sexism Inventory (ASI) [19] was used. This inventory is composed of 22 items that form two subscales (BS and HS) with 11 items each. The response format is a Likert scale with six levels ranging from 1, “strongly disagree” to 6, “strongly agree.” The items were combined in a summative way for each scale, therefore, the range of values for both subscales is from 11 to 66, with the higher values indicating greater sexism. Regarding this study, internal consistency of both subscales (BS and HS) measured by Cronbach's Alpha was 0.85 for BS and 0.93 for HS.

#### Independent variables

Pornography consumption: This information was gathered with the question “Have you consumed pornography voluntarily in the last year?” The response categories were: “Yes, I have consumed pornography in the last year/No, I have not consumed pornography in the last year”.

Sexual orientation responses were collected using the following question: “Which of the following affirmations do you feel most identified with?”; “I am only attracted to women/ I am normally attracted to women but sometimes I am attracted to men/ I am attracted to both women and men/ I am normally attracted to men, but sometimes I am attracted to women/ I am only attracted to men/ I am not attracted to men nor women/no response”. Those who reported being exclusively attracted to people of the same sex were included in the homosexual category (gay/lesbian, according to sex). Those who reported only being attracted to people of the opposite sex were classified as heterosexual. Those who reported being attracted to people of the same and opposite sex were classified as bisexual. Those who responded not being attracted to any sex (*n* = 6) were considered missing values.

### Sex: man/woman

#### Covariates

Covariates previously associated with sexism in literature are included [21].

Socio-economic variables: age (continuous variable), highest completed level of studies (no studies or primary education/secondary education or higher education), birth country (Spain/abroad). The birth country variable includes the category “abroad” for all those of Latin American origin.

### Analysis

First of all, the distribution of the variables in the sample is described, as well as the median values (Md) and Interquartile Range (IQR) of HS and BS in the total sample and stratified by sex, according to the independent variables and covariates described above.

The differences in the median values of sexism (HS and BS) between the categories of the analyzed variables were assessed using the Wilcoxon rank-sum test for two-category comparisons. For the sexual orientation variable, which encompasses four categories, the Kruskal–Wallis test followed by a post hoc Dunn's test was used. To contrast the relationship between age and sexism, the Spearman Rank correlation was employed.

Although preliminary analyses were conducted using nonparametric tests to address the non-normal distribution typically associated with Likert scale data, we opted for linear regression analysis in our main study. This decision was based on the broader acceptance of linear regression in our field, coupled with the metric nature of our summed Likert scale variables, which are conducive to parametric analysis. Additionally, our exploratory graphical analysis and the large sample size of 2,346 participants indicated that the distribution of our data was adequately normal for the purposes of linear regression.

For each dependent variable (HS and BS), a crude regression analysis was initially conducted, followed by three multiple linear regression models: a) total sample; b) women; and c) men. Due to the presence of heteroscedasticity in the residuals of the prediction for the two types of sexism, identified by the Breusch-Pagan test, robust standard errors were calculated in the estimation of the models [32]. The non-existence of multicollinearity between the variables was analyzed using the Variance Inflation Factor (VIF) test. Statistical software used was Stata 16 [33]. Given the presence of interactions with the sex variable, the models were stratified by sex.

## Results

The analyzed sample included a total of 2,346 people (missing value = 169; 6.7%) with an average age of 27.5 (SD = 4.6). The median values for HS and BS were significantly higher for men (HS: Md = 30, IQR = 20; BS: Md = 26, IQR = 17) than women (HS: Md = 20, IQR = 16; BS: Md = 21, IQR = 14). 48.5% of women and 88% of men referred to having consumed pornography intentionally in the last 12 months.

Median values of HS (Table 1) are significantly higher in people who have consumed pornography in the last year (*p* < 0.001) regarding those who have not (25 vs 23), and this is lower in homosexuals and bisexuals (*p* < 0.001) when compared to heterosexuals (18 vs 27). The Dunn’s test identified a lower HS in bisexual women (Md=16) than heterosexual women (Md=21). There were differences in all categories regarding men. Lower levels of HS were identified in homosexual men (Md=18), followed by bisexual men (Md=25) and at the highest point heterosexual men (Md=33).

**Table 1:** Distribution of hostile sexism according to sociodemographic, sexual orientation and pornography consumption variables

**Hostile sexism**	**Total sample**					**Women**					**Men**				
	**n**	**%**	**Md SH**	**IQR**	***p***	**n**	**%**	**Md SH**	**IQR**	***p***	**n**	**%**	**Md SH**	**IQR**	***p***
**Sex**					< 0.001										
Man	1169	49.83	30	20				-	-				-	-	
Women	1177	50.17	20	16				-	-				-	-	
**Pornography consumption, last 12 months**					< 0.001					< 0.01					0.08
Yes	1600	68.2	25	21		571	48.5	21	19		1029	88.0	33	16	
Non	746	31.8	23	20		606	51.5	19	15		140	12.0	30	21	
**Sexual orientation**					< 0.001					< 0.001					< 0.001
Homo/bisexual	574	24.47	18	17		306	26	16	15		268	22.9	22	19	
Heterosexual	1772	75.53	27	21		871	74	21	18		901	77.1	33	19	
**Sexual orientation**					< 0.001					< 0.001					< 0.001
Gay	119	5.1	18	16	a			-	-		119	10.2	18	16	a
Lesbian	25	1.1	19	18	a	25	2.1	19	18	a. b			-	-	
Bisexual	430	18.3	18	17	a	281	23.9	16	13	a	149	12.7	25	20	b
Heterosexual	1772	75.5	27	21	b	871	74.0	21	18	b	901	77.1	33	19	c
**Highest completed level of studies**					< 0.001					< 0.001					< 0.001
Primary	714	30.43	31	21		321	27.3	26	19		393	33.6	34	18	
Secondary or higher	1632	69.57	22	19		856	72.7	18	15		776	66.4	28	20	
**Birth country**					< 0.01					< 0.001					0.08
Abroad (Latin American)	280	11.94	28	21		165	14	24	20		115	9.8	32	26	
Spain	2066	88.06	24	20		1.012	86	19	16		1.054	90.2	30	20	
**Age: correlation (*p*)**	2346	0.07 (*p* < 0.001)				1.177	0.13 (*p* < 0.001)				1.169	0.03 (*p* = 0.270)			

1) The Wilcoxon rank-sum test was used for general contrast between two groups; 2) Spearman rank correlation was employed to examine the relationship between age and hostile sexism; 3) For variables with more than two categories, the Kruskal–Wallis test, followed by post hoc analysis with the Dunn test for pairwise comparisons, was conducted. In these cases, groups with significantly different median values are identified with different letters, while the same letters indicate no statistically significant differences between categories as determined by the Dunn test

*Abbreviations*: *HS* Hostile sexism, *Md* Median, *IQR* Interquartile Range

The median values of BS (Table 2) are significantly lower in people who have consumed pornography in the last 12 months (*p* < 0.05) regarding those who have not (23 vs 24), and homosexual or bisexual men and women (*p* < 0.001) when compared to heterosexuals (20 vs 25). The Dunn’s test identified a lower BS in bisexual women (Md=19) than heterosexual women (Md=21). Regarding men, there were differences between categories according to sexual orientation which are significant (*p* < 0.001), specifically a significantly lower BS was observed in homosexual and bisexual (Md=20) men regarding heterosexual men (Md=28).

**Table 2:** Distribution of benevolent sexism according to sociodemographic, sexual orientation and pornography consumption variables

**Benevolent Sexism**	**Total sample**					**Women**						**Men**				
	**n**	**%**	**Md SB**	**IQR**	***p***	**n**	**%**	**Md SB**	**IQR**	***p***	**n**		**%**	**Md SB**	**IQR**	***p***
**Sex**					< 0.001											
Man	1169	49.8	26	17				-	-					-	-	
Women	1177	50.2	21	14				-	-					-	-	
**Pornography consumption, last 12 months**					< 0.05					< 0.001						< 0.001
Yes	1600	68.2	23	16		571	48.5	19	11		1029		88.0	25	16	
Non	746	31.8	24	16		606	51.5	22	15		140		12.0	32	15	
**Sexual orientation**					< 0.001					< 0.001						< 0.001
Homo/bisexual	574	24.5	20	11		306	26	19	11		268		22.9	20	11	
Heterosexual	1772	75.5	25	16		871	74	21	14		901		77.1	28	16	
**Sexual orientation**					< 0.001					< 0.001						< 0.001
Gay	119	5.1	20	12	a			-	-	-	119		10.2	20	12	a
Lesbian	25	1.1	22	12	b	25	2.1	22	12	a,b				-	-	
Bisexual	430	18.3	19.5	11	a	281	23.9	19	10	a	149		12.7	20	13	a
Heterosexual	1772	75.5	25	16	b	871	74.0	21	14	b	901		77.1	28	16	b
**Highest level of studies**					< 0.001					< 0.001						< 0.001
Primary	714	30.4	26	16		321	27.3	24	15		393		33.6	29	18	
Secondary or higher	1632	69.6	23	16		856	72.7	20	12		776		66.4	25	15	
**Birth country**					< 0.001					< 0.001						< 0.05
Abroad (Latin American)	280	11.9	26	16		165	14	23	16		115		9.8	29	19	
Spain	2066	88.1	23	16		1012	86	20	13		1054		90.2	26	16	
**Age: correlation (*p*)**	2346		0.03 (*p* = 0.182)			1177		0.11 (*p* < 0.001)			1169		-0.054 (*p* = 0.067)			

1) The Wilcoxon rank-sum test was used for general contrast between two groups; 2) Spearman rank correlation was employed to examine the relationship between age and benevolent sexism; 3) For variables with more than two categories, the Kruskal–Wallis test, followed by post hoc analysis with the Dunn test for pairwise comparisons, was conducted. In these cases, groups with significantly different median values are identified with different letters, while the same letters indicate no statistically significant differences between categories as determined by the Dunn test

*Abbreviations*: *HS* Hostile sexism, *Md* Median, *IQR* Interquartile Range

**Fig. 1:**
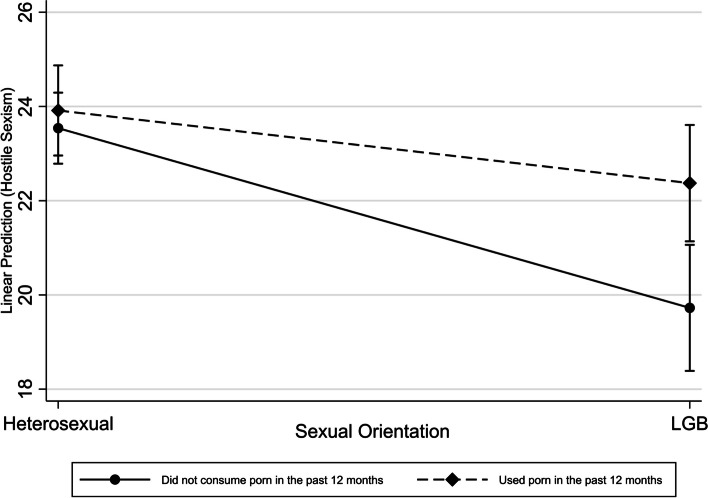
Interaction of sexual orientation and pornography consumption in women (adjusted predictions, 95% CIs)

Table 3 shows crude and adjusted estimations of variables associated with mean levels of HS. An interaction is identified in women between sexual orientation and pornography consumption (*p* < 0.05) (Fig. 1) in the mean levels of HS. Homosexual and bisexual women who did not consume pornography had, on average, lower levels of HS compared to heterosexual women, as indicated by the homosexual/bisexual coefficient [β(95%CI): -3.81(-5.36;-2.27)]. However, this negative effect on HS levels was less pronounced among homosexual/bisexual women who consumed pornography, as reflected in the positive coefficient of the interaction term [β(95%CI): 2.27(0.11; 4.43)]. This suggests that while being homosexual/ bisexual is associated with lower levels of HS compared to heterosexuals, the consumption of pornography mitigates this difference. Men who consumed pornography had significantly higher mean values of HS than those who did not [β(95%CI): 2.39(0.67; 4.10)]. Homosexual and bisexual men showed lower values of HS than heterosexual men [β(95%CI):-2.98(-4.52; -1.45)].

**Table 3:** Variables associated with hostile sexism. Linear regression model/ Multiple regression model with robust variance

**HOSTILE SEXISM**	**Linear regression**					**Multiple regression**														
	**Total sample (*n* = 2346)**					**Total sample (*n* = 2346)**					**Women (*n* = 1177)**					**Men (*n* = 1169)**				
Indicator	B		95% CI		Robust SE B	B		95% CI		Robust SE B	B		95% CI		Robust SE B	B		95% CI		Robust SE B
			LL	UL				LL	UL				LL	UL				LL	UL	
**Sex** (Reference: women)											-		-		-	-		-		-
Men	7.66	***	6.66	8.65	0.51	3.78	***	2.85	4.70	0.47										
**Pornography consumption, last 12 months** (reference: non)																				
Yes	1.92	**	0.83	3.01	0.55	1.38	**	0.50	2.27	0.45	0.38		-0.85	1.60	0.62	2.39	**	0.67	4.10	0.87
**Sexual orientation** (reference: heterosexual)																				
Homo/Bisexual	-5.9	***	-7.1	-4.8	0.58	-2.66	***	-3.59	-1.73	0.47	-3.81	***	-5.36	-2.27	0.79	-2.98	***	-4.52	-1.45	0.78
**Highest level of studies** (reference: primary)																				
Secondary or higher	-5.3	***	-6.4	-4.1	0.57	-2.61	***	-3.53	-1.68	0.47	-3.05	***	-4.30	-1.81	0.63	-2.16	**	-3.52	-0.80	0.69
**Birth country** (reference: Spain)																				
Abroad (Latin America)	2.45	**	0.77	4.13	0.86	0.70		-0.64	2.05	0.68	0.60		-1.04	2.24	0.84	0.74		-1.52	2.99	1.15
**Age**	0.19	**	0.08	0.31	0.06	0.17	***	0.08	0.26	0.05	0.12	*	0.01	0.24	0.06	0.20	**	0.06	0.33	0.07
*Significant Interaction Terms*																				
Pornography * homo/bisexual											2.27	*	0.11	4.43	1.1					
Constant						26.50	***	25.55	27.45	0.49	27.26	***	26.06	28.47	0.61	29.19	***	0.95	27.32	31.07
**Model Summary:**						R^2^		F (df)		Sig	R^2^		F (df)		Sig	R^2^		F (df)		Sig
						43.2%	318.98 (7. 2338)			*p* < *0.001*	41.4%	118.52 (7. 1169)			*p* < *0.001*	35.1%	130.07 (6. 1162)			*p* < *0.001*

Multiple regression coefficients are also adjusted by "benevolent sexism"

*Abbreviations*: *95% CI* 95% Confidence Interval, *LL* Lower Limit of confidence interval, *UL* Upper Limit of confidence interval, *SE B* Standard Error of Beta, *df* Degrees of freedom

^***^ *p* < 0.001

^**^ *p* < 0.01

^*^ *p* < 0.05

Age was positively associated with HS both in women [β IC (95%): 0,12(0.01; 0.24)] and men [β IC (95%): 0,20(0.06; 0.33)], while having a higher education decreased mean levels of HS in women [β IC (95%): -3.05 (-4.30;-1.18)] and men [β IC (95%): -2.16(-3.52; -0.80)] when compared to those with secondary or lower levels of education.

Table 4 displays crude and adjusted estimations of variables associated with mean levels of BS. In the total sample, interactions were identified between sex and pornography consumption (*p* < 0.001), between sex and sexual orientation (*p* < 0.001) and between sex and age (*p* < 0.001) in mean levels of BS.

**Table 4:** Variables associated with benevolent sexism. Linear regression model/ Multiple regression model with robust variance

**BENEVOLENT SEXISM**	**Linear regression**					**Multiple regression**														
	**Total sample (*n* = 2346)**					**Total sample (*n* = 2346)**					**Women (*n* = 1177)**					**Men (*n* = 1169)**				
Indicator	B		95% CI		Robust SE B	B		95% CI		Robust SE B	B		95% CI		Robust SE B	B		95% CI		Robust SE B
			LL	UL				LL	UL				LL	UL				LL	UL	
**Sex** (Reference: women)											-		-		-	-		-		-
Men	4.37	***	3.57	5.16	0.41	4.32	***	2.82	5.82	0.77										
**Pornography consumption, last 12 months** (reference: non)																				
Yes	-1.13	*	-2.02	-0.24	0.46	-2.20	***	-3.03	-1.37	0.42	-2.16	***	-2.99	-1.32	0.42	-4.30	***	-5.75	-2.86	0.74
**Sexual orientation** (reference: heterosexual)																				
Homo/Bisexual	-4.43	***	-5.27	-3.59	0.43	0.10		-0.82	1.03	0.47	0.20		-0.73	1.14	0.48	-3.10	***	-4.21	-1.99	0.57
**Highest level of studies** (reference: primary)																				
Secondary or higher	-3.77	***	-4.67	-2.87	0.46	-0.99	**	-1.73	-0.24	0.38	-1.11	*	-2.1	-0.12	0.51	-0.79		-1.89	0.31	0.56
**Birth country** (reference: Spain)																				
Abroad (Latin America)	2.46	***	1.13	3.79	0.68	1.51	**	0.48	2.53	0.52	1.54	*	0.21	2.86	0.68	1.36		-0.24	2.96	0.82
**Age**	0.07		-0.02	0.16	0.05	0.12	*	0.02	0.21	0.05	0.11	*	0.02	0.21	0.05	-0.16	**	-0.26	-0.05	0.05
*Significant Interaction Terms*																				
Sex (man)* age						-0.27	***	-0.41	-0.1	0.07										
Sex (man)* pornography (yes)						-2.07	*	-3.72	-0.4	0.84										
Sex (man)* sexual orientation (homo/bisexual)						-3.01	***	-4.41	-1.6	0.72										
Constant						25.9	***	25.1	26.7	0.41	26.08	***	25.14	27.01	0.48	30.29	***	28.75	31.84	0.79
**Model Summary:**						R^2^		F (df)		Sig	R^2^		F (df)		Sig	R^2^		F (df)		Sig
						41.8%	148.34 (10. 2335)			*p* < *0.001*	41.2%	106.20 (6. 1170)			*p* < *0.001*	37.3%	118.61 (6. 1162)			*p* < *0.001*

Multiple regression coefficients are also adjusted by "hostile sexism"

*Abbreviations*: *95% CI* 95% Confidence Interval, *LL* Lower Limit of confidence interval, *UL* Upper Limit of confidence interval, *SE B* Standard Error of Beta, *df* Degrees of freedom

^***^ *p* < 0.001

^**^ *p* < 0.01

^*^ *p* < 0.05

**Fig. 2:**
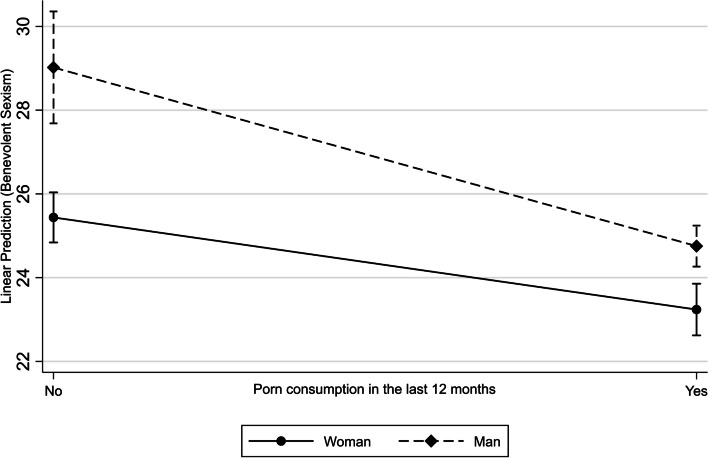
Interaction of sex and pornography consumption on benevolent sexism

**Fig. 3:**
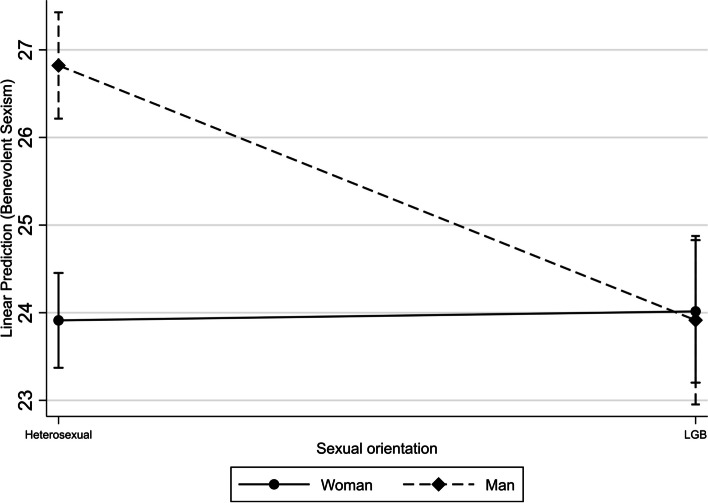
Interaction of sex and sexual orientation on benevolent sexism

**Fig. 4:**
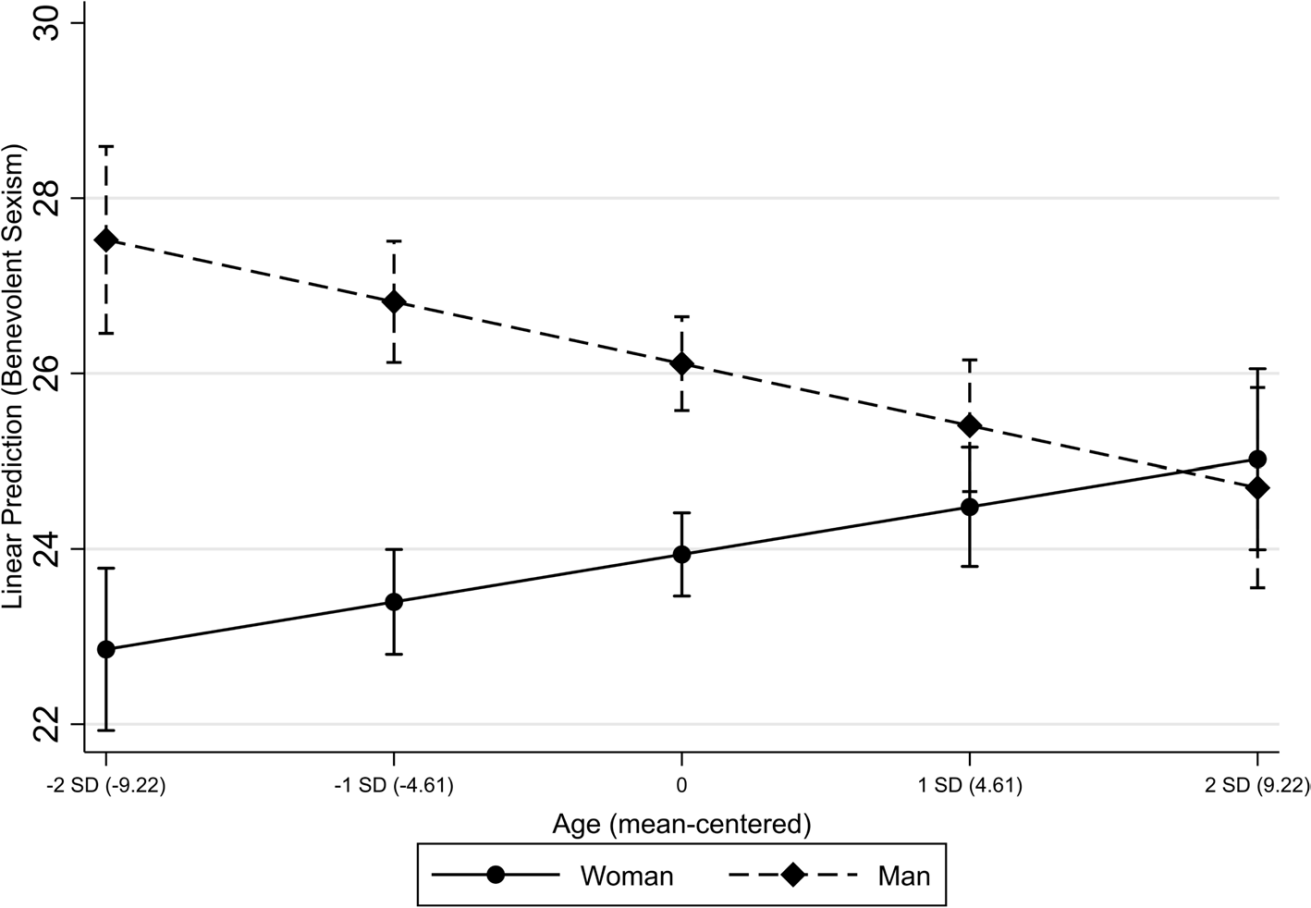
Interaction of sex and age on benevolent sexism

Regarding both women [β (95%CI): -2.16 (-2.99; -1.32)] and men [β (95%CI): -4.30 (-5.75; -2.86)], those who consume pornography have mean values of BS lower than those who do not. This effect is significantly higher in men than women (Fig. 2). Homosexual and bisexual men have mean values of BS lower than heterosexual men [β (95%CI): -3.10 (-4.21; -1.99)]. This association was not identified in women (Fig. 3). As age increases, mean levels of BS in women rise [β(95%CI): 0.11(0.02; 0.21)], while it decreases in men [β(95%CI): -0.16(-0.26; -0.05)] (Fig. 4).

Another variable associated with higher BS in women was being of Latin American origin, when compared to having been born in Spain [β(95%CI): 1.54(0.21; 2.86)]; while having a higher education regarding having secondary or lower education [β(95%CI): -1.11(-2.10; -0.12)] was associated with a lower BS.

## Discussion

The aim of this study is to analyze the association of pornography consumption and sexual orientation with HS and BS in young men and women. The results of this study partially verify the initial hypotheses. In terms of the first hypothesis, an association between sexual orientation and HS is confirmed to exist in men. That is, men with homosexual and bisexual orientation present lower HS than men with heterosexual orientation. Regarding women, this effect is exclusively observed in those who do not consume pornography. This association is confirmed between sexual orientation and BS, specifically homosexual and bisexual men displayed lower mean values of BS than heterosexual men. This effect is not observed in women.

As for the second hypothesis, the results confirm that there is a positive association between pornography consumption and HS. This association is positive regardless of sexual orientation in men. In women, the association between pornography consumption and HS is significant and positive exclusively regarding homosexual and bisexual women. In terms of BS, a negative association has been found between pornography consumption and BS, with the association being greater in men than in women. This association is regardless of sexual orientation.

### Hostile sexism

Our results show an association between pornography consumption and sexual orientation with mean levels of HS in both sexes. In line with what was previously observed [16, 34, 35], our results confirm that men who consume pornography show higher mean values of HS regardless of their sexual orientation. In relation to this result, it is important to highlight that being exposed to pornography starts at younger ages, as pornography becomes part of the construction of eroticism in adolescence [36]. The association between pornography and HS [16], as well as the association between HS and violence against women [21, 37–39], highlights the need to offer education programs that reduce sexism at younger ages [40] and to provide sex education based on pleasure and mutual respect from early ages.

Homosexual and bisexual men display lower mean levels of HS than heterosexual men. This effect is independent of the rest of the analyzed covariates. The absolute values show a graduation in the mean levels of HS according to sexual orientation from heterosexual men (higher HS) to homosexual men (lower HS). These results support the points highlighted by Cowie et al. (2019) that homosexual and bisexual men share to a lesser extent the values and attitudes of heterosexual hostility present in HS, given that they do not perceive that women could dominate them in romantic relationships [25]. Homosexual and bisexual men could also feel discriminated against due to attitudes and behaviors associated with HS that reject non-heterosexual sexual orientations [41]. In line with our results, the studies by Cowie et al. (2019) identified that heterosexual men show a higher mean HS score than homosexual and bisexual men. The mean HS values between homosexual and bisexual men did not show significant differences between them [25].

Regarding women, an interaction that has not been previously described in literature between sexual orientation and pornography consumption in mean levels of HS has been identified. Homosexual and bisexual women that consume pornography obtained mean levels of HS higher than those who did not. However, in heterosexual women, mean levels of HS show no differences regarding pornography consumption. Homosexual and bisexual women that consume pornography obtained mean levels of HS higher than those who did not. It is possible that the higher use of pornography described in homosexual and bisexual women [42, 43] compared to heterosexual women, increases the mean values of HS in this group of women when compared to those who do not consume pornography. Regarding heterosexual women, pornography consumption did not increase the mean values of HS. This last result could be related to finds of Bőthe et al. [42], who suggest heterosexual women consume pornography frequently with their partners with the main aim of improving sexual relations in a romantic relationship.

In the same sense as men, lower HS observed in our study in homosexual and bisexual women compared to heterosexual women in the group that does not consume pornography could be explained by HS discrimination towards non-heterosexual orientations [41]. As romantic relationships between women deviate from traditional understandings of appropriate female behavior and gender norms, they can be perceived as challenging male power. Therefore, homosexual and bisexual women are more likely to be targets of HS. It would then be expected for homosexual or bisexual women to express lower levels of HS as these ideologies actively marginalize them [25].

The relationship between HS and age is difficult to assess as most studies are cross-sectional with samples limited to a certain age group [44]. Our results describe a positive association between HS and age, independently of the rest of covariates in a sample that comprises late adolescence and early adulthood. Similarly [45], a positive association between AS and age is reported in university women. Young samples continue to endorse sexism as previously described in literature [21, 35, 46, 47]. A large longitudinal study (N = 10,398) conducted by Hammond et al. (2018) describes U-shaped trajectories of HS across ages [44], showing consistency with cross-sectional data from Spanish samples [48, 49], describing a high endorsement of HS in late adolescence, lower in middle adulthood, and then high again at older ages. Although our sample is young and was expected to endorse high levels of HS, we observe an increase with age, contrary to study by Hammond et al. (2018), in which endorsement of HS decreases in young cohorts [44].

### Benevolent sexism

The association between pornography consumption and sexual orientation with BS is more complex than HS [25]. Our results show that pornography consumption is associated with lower mean levels of BS both in men and women, although this effect is significantly higher in men. Mainstream pornography shows images far from BS behaviors and attitudes, as the majority eroticize violent and hostile behavior by exposing women as a sexual object for male pleasure [5]. The frequency of pornography consumption is higher among men than women [8, 12, 13], as well as consuming pornography with violent content [50, 51]. These differences could explain the differences observed in the mean levels of BS among men and women that consume pornography.

Homosexual and bisexual men displayed a lower BS than heterosexual men. Heterosexual intimacy appears in BS by means of subtle behaviors that respond to the perception of women as weak, vulnerable beings that need to be protected and cared for by men, thus forming a hierarchy between men and women. It is possible that the lower BS observed in homosexual and bisexual men, when compared to heterosexual men, is due to the fact that BS does not offer privileges to homosexual and bisexual men as they do not need gratitude or recognition from women towards them. Regarding women, mean levels of BS remained constant regardless of sexual orientation. Given that BS is projected through behaviors and attitudes that could be positively reinterpreted, it is possible that women, regardless of their sexual orientation, accept the apparent privileges that BS offers [52, 53].

The higher mean levels of BS observed in women born abroad (Latin America), when compared to women born in Spain, have previously been described in literature [45, 54, 55]. Recently published studies [55] argue that Latin American migrant adolescents adhering to sexist attitudes is a reflection of the existing gender inequality in each country. Other authors suggest that women supporting attitudes associated with BS is an adaptive response to insecure and hostile environments [56, 57], such as migration.

Regardless of the other covariates and in accordance with previous studies [48, 58–60], people with university studies have lower AS levels than people with lower levels of education, mainly in its hostile dimension in both sexes and in the benevolent dimension in women. Given the cross-sectional nature of the data, we do not know if less sexist people have greater opportunities to reach higher levels of education or if education and the interpersonal relationships that are established around them make people have lower levels of AS. However, educational interventions aimed at reducing sexist attitudes have shown to be effective for adolescents [40], therefore demonstrating the essential role education has in fighting for equality between men and women [61–63].

The results of this study must be interpreted taking into consideration the limitations. Collecting information on pornography consumption does not enable us to characterize the frequency of consumption or the type of content that is viewed. Frequency of consumption, type of content and starting age of porn consumption are variables that should be considered for future research. These variables could be associated with sexual orientation [42, 43] and mean values of sexism [25, 26]. Although we are lacking this information, our results identify an association between last’s year pornography consumption and sexism. However, the sample size did not have enough statistical power to independently analyze homosexual and bisexual men and women. An association between country of birth and BS is identified in women. Nonetheless, we do not know the time spent living in Spain, which would be a valuable variable to include in order to better describe and understand this association. The cross-sectional nature of the design does not allow us to establish the temporality of the association between pornography and sexism, and an inverse directionality between both variables cannot be ruled out. We do not know if the self-reported pornography consumption can be exposed to a bias of social undesirability and if so, we do not know if this bias is or is not a differential bias according to the sex or sexual orientation of the participants. The fact that the information is collected anonymously online and without the physical presence of an interviewer, we believe may have minimized such bias, if it exists.

## Conclusions

Despite these limitations, our results reveal new information on a subject that has not been studied much yet and is of great importance at present. Our results allow us to confirm that there is an association between pornography consumption and sexual orientation with AS. As sexism is the substratum of inequality between men and women and, in turn, it is associated with violence against women and homophobia/biphobia, it is urgent to launch affective-sexual education programs for young people that take into account the social determinants of sexism, such as level of education, place of origin or sexual orientation. Given the social component of AS, it is also necessary to promote strategies at the structural level so that equality between men and women permeates the social construction of pleasure and sexual desire.

## Data Availability

The datasets and material that have been produced during the current study are available from the corresponding author on reasonable request that guarantees their use according to the ethical procedures adopted in this project and participants’ informed consent documents content.

## Abbreviations

BS: Benevolent sexism
HS: Hostile sexism
AS: Ambivalent sexism
SV: Sexual violence
AS: Ambivalent sexism inventory
Md: Median
VIF: Variance inflation factor
SD: Standard deviation
95% CI: 95% Confidence Interval
LL: Lower Limit of confidence interval
UL: Upper Limit of confidence interval
SE B: Standard Error of Beta
df: Degrees of freedom
